# Impact of multisite colonization and comparative microbiological outcomes of colistin-sulbactam vs. colistin-tigecycline in CRAB hospital-acquired pneumonia: a propensity score-matched analysis

**DOI:** 10.3389/fcimb.2026.1804539

**Published:** 2026-06-04

**Authors:** Shiyu Liao, Lan Shen

**Affiliations:** 1 Department of Respiratory, Yueyang Municipal Hospital of Hunan Normal University, Yueyang, Hunan, China; 2 Department of Obstetrics, Yueyang Municipal Hospital of Hunan Normal University, Yueyang, Hunan, China

**Keywords:** carbapenem-resistant Acinetobacter baumannii, colistin, hospital-acquired pneumonia, multisite colonization, sulbactam

## Abstract

**Background:**

The optimal combination therapy for carbapenem-resistant *Acinetobacter baumannii* (CRAB) pneumonia remains controversial. This study aimed to compare the microbiological efficacy, clinical outcomes, and safety of colistin-sulbactam versus colistin-tigecycline regimens in patients with CRAB hospital-acquired or ventilator-associated pneumonia (HAP/VAP).

**Methods:**

A single-center, retrospective cohort study was conducted from 2019 to 2024. Adult patients with culture-confirmed CRAB HAP/VAP receiving either colistin-sulbactam (Group A) or colistin-tigecycline (Group B) for ≥72 hours were included. Propensity score matching (PSM) was utilized to balance baseline covariates, including APACHE II scores and renal function. The primary endpoint was microbiological eradication at Day 7; secondary endpoints included 28-day all-cause mortality, clinical cure, and adverse events.

**Results:**

Among 176 eligible patients, 42 matched pairs (n=84) were generated. The colistin-sulbactam group achieved significantly higher microbiological eradication rates at Day 7 compared to the colistin-tigecycline group (66.7% vs. 40.5%; P = 0.013). Subgroup analysis revealed that this benefit was particularly pronounced in patients with extensively drug-resistant (XDR) strains (OR 4.75; 95% CI 1.58–14.26) and those with multisite colonization. Clinical cure rates were also higher in Group A (59.5% vs. 35.7%; P = 0.029). No significant difference was observed in 28-day all-cause mortality (31.0% vs. 38.1%; P = 0.490).

**Conclusion:**

Colistin combined with high-dose sulbactam demonstrated higher microbiological clearance and clinical cure rates compared to colistin-tigecycline, particularly in patients with XDR strains and high bacterial burden. The sulbactam-based regimen offers a favorable efficacy and safety profile and should be considered a preferred therapeutic strategy for severe CRAB infections.

## 1 Introduction

Carbapenem-resistant *Acinetobacter baumannii* (CRAB) has emerged as a formidable global health threat, particularly as a leading cause of hospital-acquired pneumonia (HAP) and ventilator-associated pneumonia (VAP) in critically ill patients (Hammoudi Halat and Ayoub Moubareck, 2024). The bacterium’s extensive antibiotic resistance severely limits therapeutic options, contributing to high morbidity and mortality rates, which can exceed 60% in patients with CRAB-associated VAP (Hammoudi Halat and Ayoub Moubareck, 2024; Najafabadi and Soltani, 2024). This public health crisis has been exacerbated by the COVID-19 pandemic, which saw an increase in antimicrobial misuse and a surge in VAP incidence, further fueling the spread of multidrug-resistant organisms like CRAB (Rangel and De-Simone, 2024). Consequently, the World Health Organization (WHO) has designated CRAB as a “critical” priority pathogen, underscoring the urgent need for novel and effective treatment strategies (Choi and Kim, 2024).

For decades, colistin, a polymyxin antibiotic, has been repurposed as a last-resort therapy for CRAB infections. However, its utility is hampered by significant drawbacks, including a high incidence of nephrotoxicity, suboptimal penetration into the lung parenchyma, and the emergence of resistance (Serapide et al., 2024). Clinical trials have failed to demonstrate a consistent benefit of colistin-based combination therapy over monotherapy, particularly when combined with meropenem, leading major infectious diseases societies to recommend against this specific pairing (Choi and Kim, 2024). This therapeutic impasse has driven the exploration of alternative combination regimens to enhance efficacy and mitigate toxicity. Among the most discussed are combinations of colistin with either sulbactam or tigecycline.

The colistin-sulbactam combination is founded on a strong synergistic rationale. Colistin disrupts the bacterial outer membrane, facilitating the entry of sulbactam, which possesses intrinsic bactericidal activity against *A. baumannii* through binding to penicillin-binding proteins (Han et al., 2019). Recent evidence supports this approach, with a 2024 randomized controlled trial demonstrating that high-dose sulbactam (12 g/day) combined with colistin significantly improved microbiological cure and showed a trend towards lower 28-day mortality compared to a lower-dose regimen for CRAB pneumonia (Ungthammakhun et al., 2024). Recent literature and narrative reviews increasingly favor high-dose sulbactam-based regimens as a cornerstone of CRAB therapy (Serapide et al., 2024).

In contrast, the colistin-tigecycline combination, while also exhibiting *in vitro* synergy, is mired in controversy. Tigecycline’s utility is severely limited by its poor pharmacokinetic profile, characterized by low serum and lung concentrations, which may be insufficient to treat severe systemic infections (Choi and Kim, 2024). More alarmingly, the U.S. Food and Drug Administration (FDA) has issued a black box warning for tigecycline, citing an increased all-cause mortality risk, a risk that is most pronounced in patients treated for VAP (US Food and Drug Administration, 2010). Furthermore, real-world data have linked tigecycline to a significant risk of coagulation dysfunction, particularly hypofibrinogenemia (Guo et al., 2022). Retrospective studies have failed to show a significant mortality benefit when adding standard-dose tigecycline to colistin for CRAB bacteremia, raising further questions about its clinical value in severe infections (Choi and Kim, 2024).

Despite the distinct and compelling arguments for and against these two colistin-based combination regimens, a critical evidence gap persists. A systematic review of recent literature reveals a striking lack of head-to-head clinical trials directly comparing the efficacy and safety of colistin-sulbactam versus colistin-tigecycline for the treatment of CRAB-HAP/VAP (Wang et al., 2021; Serapide et al., 2024). This absence of comparative data leaves clinicians without high-level evidence to guide treatment decisions, perpetuating uncertainty in managing this life-threatening infection.

Therefore, the objective of the present study is to directly compare the clinical outcomes, including efficacy and safety, of a colistin-sulbactam combination versus a colistin-tigecycline combination in patients with microbiologically confirmed HAP or VAP caused by CRAB. We hypothesized that colistin-sulbactam would demonstrate higher microbiological eradication rates and that multisite colonization, serving as a clinical proxy for high systemic bacterial burden, would act as a critical independent predictor of microbiological failure and potentially modulate the comparative effectiveness of these antimicrobial regimens. This research aims to provide crucial evidence to address existing therapeutic uncertainty and inform clinical practice for this vulnerable patient population.

## 2 Methods

### 2.1 Study design and setting

This single-center, retrospective cohort study was conducted at the Department of Critical Care Medicine of *, a tertiary care teaching hospital, utilizing data integrated from the Electronic Medical Record (EMR) and Laboratory Information Systems (LIS) from January 1, 2019, to December 31, 2024. The primary objective was to compare the microbiological outcomes of colistin-sulbactam versus colistin-tigecycline combination therapies in patients with CRAB-associated HAP. Specifically, we sought to test the hypothesis that multisite colonization significantly impacts treatment success, evaluating its role as a key independent determinant of microbiological eradication within our comparative analysis.

### 2.2 Study population

Participants were recruited from the aforementioned database through a multistage screening process. All adult inpatients admitted during the study period with a positive respiratory tract culture (sputum or bronchoalveolar lavage fluid [BALF]) for *A. baumannii* were initially identified. Potential participants then underwent a rigorous three-step validation: (1) automated filtration to isolate strains with confirmed carbapenem resistance, defined as a minimum inhibitory concentration (MIC) of meropenem or imipenem ≥8 mg/L, which corresponds to the Clinical and Laboratory Standards Institute (CLSI) resistance breakpoint (CLSI M100) (Clinical and Laboratory Standards Institute (CLSI), 2023); (2) manual chart review by two independent infectious disease specialists to verify the pneumonia diagnosis and treatment regimen details; and (3) final adjudication by a senior consultant for cases with diagnostic ambiguity. Discrepancies regarding eligibility were resolved through consensus discussion.

Eligibility was determined based on strict adherence to the following criteria. Inclusion criteria comprised all of the following: (1) Age ≥18 years; (2) Confirmed diagnosis of HAP or VAP according to the 2016 IDSA/ATS clinical practice guidelines, defined as a new lung infiltrate plus clinical evidence of infectious origin (e.g., new onset of fever, purulent sputum, leukocytosis, or decline in oxygenation) occurring ≥48 hours after admission or endotracheal intubation (Kalil et al., 2016); (3) Microbiologically confirmed CRAB identified as the predominant causative pathogen, defined by quantitative culture thresholds (≥10^4^ CFU/mL for BALF or ≥10^6^ CFU/mL for sputum/endotracheal aspirate) or semi-quantitative growth of ≥3+ with consistent Gram stain morphology in the absence of other potential pathogens; (4) Receipt of targeted combination therapy with either intravenous colistimethate sodium (CMS) plus sulbactam (Group A) or CMS plus tigecycline (Group B), initiated or adjusted specifically based on susceptibility reports that included results for colistin and the respective companion drug, and continued for ≥72 hours (Tamma et al., 2023).

Exclusion criteria encompassed any of the following: (1) Polymicrobial infections where other clinically significant pathogens (e.g., *Pseudomonas aeruginosa*, methicillin-resistant *Staphylococcus aureus* [MRSA], *Stenotrophomonas maltophilia*, or other Enterobacterales such as *Klebsiella pneumoniae*, among others) were concomitantly isolated from the same respiratory sample, which could confound the attribution of clinical outcomes; (2) Death or discharge within 48 hours after the initiation of antibiotic therapy; (3) Incomplete medical records, specifically the absence of baseline renal function data (serum creatinine) or the inability to assess multisite colonization status due to the lack of any non-respiratory culture data (from sites such as rectal swabs, skin, urine, or blood) within 7 days prior to the diagnosis of pneumonia; (4) Pregnancy or lactation; (5) Transfer to another facility during the treatment course resulting in unknown survival status; (6) Patients with documented severe hepatic impairment (Child-Pugh Class C); (7) Patients initially treated with colistin-sulbactam who received a standard or low daily dose of the sulbactam component (defined as < 4 g/day), as this study specifically aimed to evaluate the high-dose regimen.

### 2.3 Variables and data collection

Data extraction was performed by trained research coordinators using a standardized electronic case report form (eCRF). Baseline demographic and clinical characteristics were collected at the time of hospital admission and infection onset, including age, gender, body mass index (BMI), and admission source (community vs. transfer from other facilities). Comorbidities were quantified using the Charlson Comorbidity Index (CCI) (Charlson et al., 1987), while the severity of illness at the onset of HAP/VAP was assessed using the Acute Physiology and Chronic Health Evaluation (APACHE) II score (Knaus et al., 1985) and the Sequential Organ Failure Assessment (SOFA) score (Vincent et al., 1996).

To evaluate the impact of colonization burden, the multisite carriage status was assessed based on all available clinical cultures obtained from non-respiratory sites within 7 days prior to the diagnosis of pneumonia. According to our institutional infection control policy, active surveillance cultures for multidrug-resistant organisms are routinely mandated for all critically ill patients upon admission to the Department of Critical Care Medicine and weekly thereafter. Conversely, for non-critically ill patients initially managed in general wards, non-respiratory cultures were primarily symptom-driven rather than routine. Commonly screened or clinically sampled sites included the axillary/inguinal skin, rectum, urine, blood, and vascular catheter tips. Patients were stratified into three groups: (1) Non-colonized (absence of *A. baumannii* in any non-respiratory culture); (2) Single-site colonization (positive culture at only one non-respiratory site); and (3) Multisite colonization (positive cultures at ≥2 non-respiratory sites), serving as a proxy for total body bacterial burden (Latibeaudiere et al., 2015). Antibiotic exposure was defined as the administration of systemic antimicrobials for ≥48 hours within the 90 days preceding the current infection. Special attention was paid to prior carbapenem exposure, specifically stratifying patients based on the verified use of meropenem or imipenem (Yes/No). For the purpose of subgroup analysis, extensively drug-resistant (XDR) *A. baumannii* was defined according to the international consensus proposal by Magiorakos et al. (2012), as non-susceptibility to at least one agent in all but two or fewer antimicrobial categories (i.e., bacterial isolates remaining susceptible to only one or two antimicrobial categories).

Potential confounders influencing clinical outcomes were comprehensively recorded to minimize selection bias. In addition to general demographics, these included: Physiological reserves and nutritional status: Baseline estimated glomerular filtration rate (eGFR, calculated via the CKD-EPI 2021 equation) and serum albumin levels (<30 g/L defined as hypoalbuminemia), which significantly alter the pharmacokinetics of tigecycline and colistin (Inker et al., 2021); (2) Treatment-related factors: Presence of invasive devices (central venous catheters, urinary catheters), requirement for mechanical ventilation, and vasopressor support for septic shock at the initiation of therapy; (3) Host immune status: Presence of immunosuppression, defined as active malignancy, chemotherapy, or long-term corticosteroid use.

### 2.4 Treatment regimens

Antimicrobial therapy was standardized according to institutional protocols aligned with the international consensus guidelines for the optimal use of polymyxins (Tsuji et al., 2019). All patients received intravenous CMS as the backbone of the combination regimen. To rapidly achieve therapeutic plasma concentrations, a loading dose of 300 mg colistin base activity (CBA) (equivalent to approximately 9 million IU) was administered within the first hour of therapy. The maintenance regimen was initiated 12 hours after the loading dose. For patients with normal renal function (creatinine clearance ≥80 mL/min), the maintenance dose was 150 mg CBA every 12 hours. For patients with renal impairment (creatinine clearance <80 mL/min), the maintenance dose was calculated daily based on the most recent serum creatinine value, using the dosing nomogram recommended by the consensus guidelines (Tsuji et al., 2019), which correlates dose with creatinine clearance and body weight to mitigate nephrotoxicity.

The combination agents were administered based on the allocated treatment group. Patients in Group A received a high-dose sulbactam regimen via either ampicillin-sulbactam or cefoperazone-sulbactam. The target daily dose of the sulbactam component was ≥4 g, with the actual administered doses ranging from 4.5 to 9.0 g per day, as recommended for CRAB infections (Kalil et al., 2016). Specifically, the exact daily dose within this range was individualized based on the patient’s renal function (creatinine clearance) to mitigate the risk of neurotoxicity while ensuring therapeutic efficacy. For patients with normal or augmented renal function, doses at the higher end of the spectrum (e.g., 8.0–9.0 g/day) were targeted. To optimize the pharmacodynamic parameter (time above the minimum inhibitory concentration, %fT>MIC), the daily dose was divided and administered every 6 to 8 hours, predominantly utilizing extended intravenous infusions (e.g., over 3 to 4 hours). The daily dose of the sulbactam component was calculated according to the fixed ratio (as specified in the product information for the specific brand used) of the respective ampicillin-sulbactam or cefoperazone-sulbactam formulation. (2:1 for ampicillin-sulbactam; 1:1 or 2:1 for cefoperazone-sulbactam). Patients in Group B received tigecycline with a standard loading dose of 100 mg, followed by a maintenance dose of 50 mg every 12 hours. No dose adjustment was performed for mild-to-moderate hepatic impairment (Food and Drug Administration (FDA), 2005). In both groups, inhalation colistin was not routinely administered. The total duration of combination therapy was determined by the treating team based on clinical response and microbiological clearance, with a minimum requirement of 72 hours for study inclusion.

### 2.5 Outcomes

The primary endpoint was microbiological eradication, evaluated at two specific time intervals: Day 7 (primary assessment; window: Days 5–9) and Day 14 (test-of-cure; window: Days 12–16) after the initiation of combination therapy. To ensure objective rigor, this analysis was strictly restricted to the microbiologically evaluable population, defined as patients who had available follow-up respiratory tract cultures within these respective windows. Microbiological eradication was defined as the documented absence of the original *A. baumannii* strain in follow-up cultures. Conversely, microbiological failure was defined as the persistence or recurrence of the causative *A. baumannii* strain in any follow-up respiratory culture obtained during treatment or before the test-of-cure window. Patients who died before a follow-up culture could be obtained were analyzed separately under the mortality endpoint and were not classified as microbiological failures unless a positive respiratory culture was obtained within 7 days preceding death.

Secondary endpoints assessed clinical efficacy and survival across the entire cohort. Clinical cure was defined as the complete resolution or significant improvement of baseline signs and symptoms (e.g., fever, purulent sputum, leukocytosis) and radiological findings, assessed at the End of Treatment (EOT) or hospital discharge (whichever occurred first), such that antibiotics were discontinued without the requirement for rescue therapy or relapse within the study period. All-cause mortality was assessed at 28 days following the first dose of the study drug. Treatment failure was defined as a composite clinical outcome by Day 28, comprising: (1) all-cause death; or (2) lack of clinical improvement (defined as no resolution of ≥2 core symptoms such as fever, leukocytosis, or oxygen requirement) or clear worsening leading to escalation of therapy (defined as the addition of a new anti-Gram-negative agent active against CRAB or the initiation of salvage therapy such as inhaled colistin). Notably, patients with microbiological persistence in the absence of clinical deterioration were not classified as clinical treatment failures.

Safety assessments focused on nephrotoxicity and other treatment-related adverse events. Acute Kidney Injury (AKI) was graded strictly according to the Kidney Disease: Improving Global Outcomes (KDIGO) clinical practice guidelines (Kidney Disease: Improving Global Outcomes (KDIGO) Acute Kidney Injury Work Group, 2012). AKI was defined as an increase in serum creatinine (SCr) by ≥0.3 mg/dL (≥26.5 µmol/L) within 48 hours or an increase in SCr to ≥1.5 times baseline. Baseline SCr was defined as the most recent value within 7 days prior to the initiation of colistin therapy. Staging was classified as: Stage 1 (1.5–1.9 times baseline SCr or increase ≥0.3 mg/dL); Stage 2 (2.0–2.9 times baseline SCr); and Stage 3 (3.0 times baseline SCr, or increase in SCr to ≥4.0 mg/dL, or initiation of renal replacement therapy). Neurotoxicity was defined as the development of new-onset neurological signs (e.g., paresthesia, dizziness, muscle weakness, or confusion) and/or the occurrence of these signs leading to a documented clinical note attributing them to colistin therapy, leading to dose reduction or discontinuation of colistin as noted by the treating physician. Hepatotoxicity (ALT/AST >3 times the upper limit of normal) was also recorded.

### 2.6 Statistical analysis

Statistical analyses were conducted using SPSS version 26.0 (IBM Corp, Armonk, NY, USA) and R version 4.3.2 (R Foundation for Statistical Computing, Vienna, Austria). The normality of continuous variables was assessed using the Kolmogorov-Smirnov test; they are presented as mean ± standard deviation (SD) or median and interquartile range (IQR). Categorical variables are summarized as frequencies and percentages. Missing data were handled via complete-case analysis (<5% missing) or multiple imputation by chained equations (MICE) (5–20% missing) to preserve statistical power. Propensity score matching (PSM) was performed to mitigate selection bias. Propensity scores were estimated via multivariate logistic regression using clinically relevant covariates: age, CCI, APACHE II score, mechanical ventilation, septic shock, immunosuppression, multisite carriage, prior meropenem exposure, and baseline renal function (eGFR). A 1:1 nearest-neighbor matching algorithm without replacement (caliper width: 0.02) matched the colistin-sulbactam and colistin-tigecycline groups. Covariate balance was confirmed with standardized mean differences (SMD) <0.1. Within the matched cohort, continuous variables were compared using paired t-tests or Wilcoxon signed-rank tests, while categorical variables were compared using McNemar’s test. Survival probabilities were estimated with the Kaplan-Meier method and compared using the stratified log-rank test. Furthermore, because mortality precludes observing microbiological clearance, time to eradication was evaluated via a competing risk analysis. The cumulative incidence function (CIF) and Gray’s test were employed, treating all-cause mortality as the competing event. A pre-specified subgroup analysis evaluated extensively drug-resistant Acinetobacter baumannii (XDR-AB) infections. All statistical tests were two-sided, with a P-value <0.05 defined as statistically significant.

## 3 Results

### 3.1 Patient selection and baseline characteristics

From 368 patients screened with A. baumannii respiratory cultures, 192 were excluded: 78 for polymicrobial infections, 45 for therapy <72 hours, 36 for incomplete baseline data, 18 for death/discharge within 48 hours, and 15 for other ineligibility. The pre-matched cohort comprised 176 patients: 92 in Group A (Colistin-Sulbactam) and 84 in Group B (Colistin-Tigecycline). Significant baseline imbalances existed, reflecting non-randomized treatment assignment. Group B exhibited greater disease severity (APACHE II; SOFA, P = 0.005), higher vasopressor requirement (P = 0.013), worse renal function (eGFR, P<0.001), and a higher multisite colonization burden (P = 0.038).

**Figure 1:**
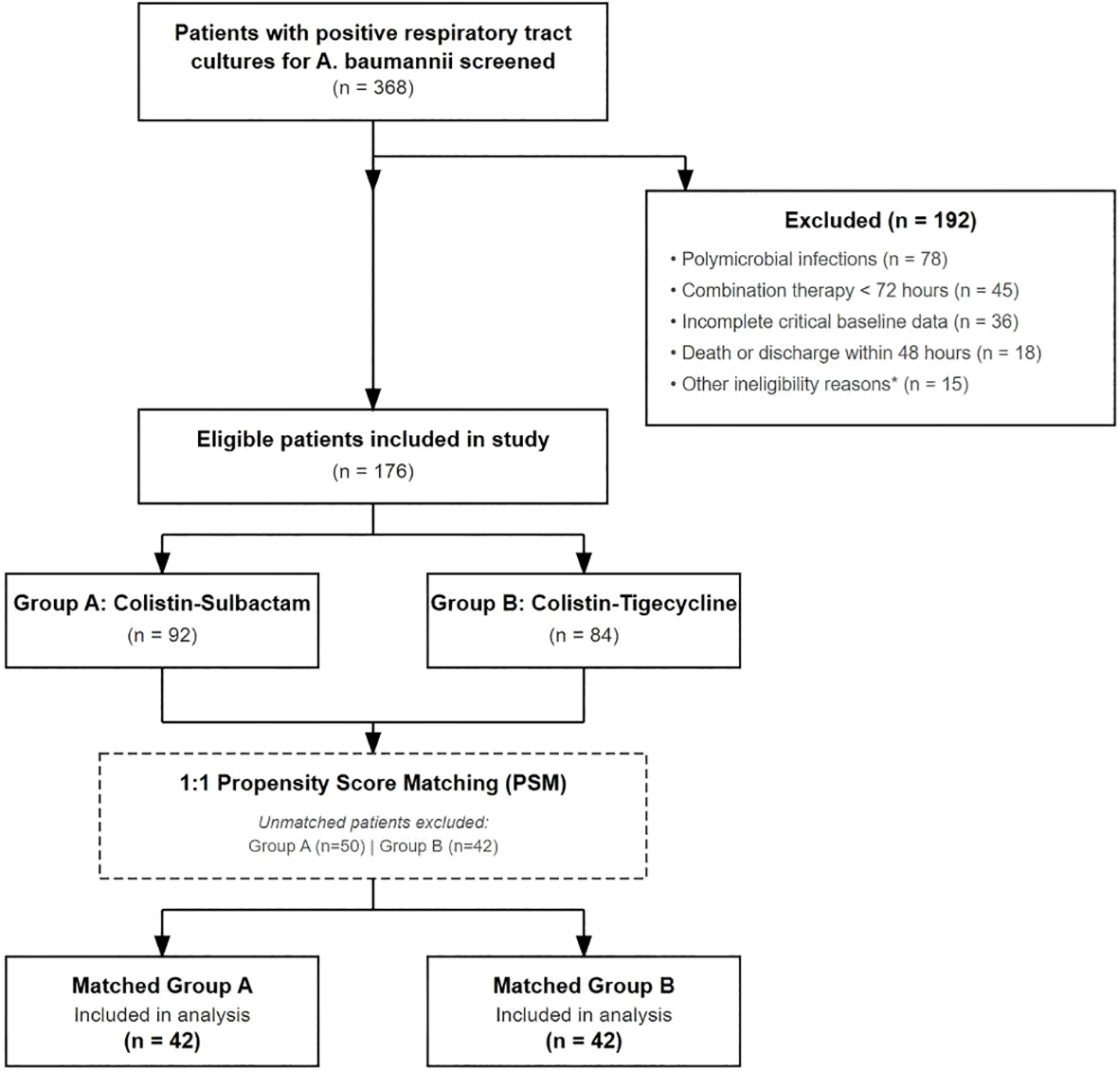
Flowchart of patient selection.

Following 1:1 nearest-neighbor PSM, 42 well-matched pairs (n=84) were generated. In this post-matched cohort, all SMD fell below 0.10, with no statistically significant differences observed across baseline variables, including XDR strain distribution and invasive device utilization (all P>0.05). This confirms that selection bias was effectively minimized, establishing a comparable foundation for clinical outcome analysis (**Figure 1**, **Table 1**).

**Table 1:** Baseline demographic and clinical characteristics of patients in Colistin-Sulbactam and Colistin-Tigecycline groups before and after propensity score matching.

Characteristic	Pre-matched cohort					Post-matched cohort			
	Group A (Col-Sul) (n=92)	Group B (Col-Tig) (n=84)	Stat. (t/χ2)	P-value	SMD	Group A (Col-Sul) (n=42)	Group B (Col-Tig) (n=42)	Stat. (t/χ2)	P-value
Demographics									
Age, years	64.2 ± 13.5	66.8 ± 12.9	1.3	0.194	0.19	65.5 ± 12.1	66.1 ± 12.4	0.22	0.824
Male sex, n (%)	58 (63.0)	55 (65.5)	0.11	0.735	0.05	27 (64.3)	28 (66.7)	0.05	0.818
BMI, kg/m²	23.5 ± 3.8	23.1 ± 4.1	0.67	0.502	0.1	23.4 ± 3.6	23.2 ± 3.9	0.24	0.808
Transfer Admission, n (%)	35 (38.0)	40 (47.6)	1.66	0.197	0.19	18 (42.9)	19 (45.2)	0.05	0.827
Comorbidities									
CCI score	4.1 ± 2.0	4.6 ± 2.3	1.54	0.126	0.23	4.4 ± 2.1	4.5 ± 2.2	0.21	0.834
Immunosuppression, n (%)	14 (15.2)	18 (21.4)	1.13	0.288	0.16	8 (19.0)	9 (21.4)	0.07	0.789
Severity & Support									
APACHE II score	20.8 ± 5.9	24.1 ± 6.2	3.61	<0.001	0.54	23.2 ± 5.8	23.5 ± 6.0	0.23	0.817
SOFA score	8.2 ± 3.1	9.6 ± 3.5	2.81	0.005	0.42	9.1 ± 3.2	9.3 ± 3.3	0.28	0.779
Septic shock, n (%)	38 (41.3)	49 (58.3)	5.04	0.025	0.34	22 (52.4)	23 (54.8)	0.05	0.827
Vasopressor support, n (%)	42 (45.7)	54 (64.3)	6.13	0.013	0.38	24 (57.1)	25 (59.5)	0.05	0.825
Mech. Ventilation, n (%)	68 (73.9)	71 (84.5)	2.96	0.085	0.26	34 (81.0)	33 (78.6)	0.08	0.782
Invasive Devices, n (%)									
- Central Venous Catheter	81 (88.0)	78 (92.9)	1.12	0.289	0.17	39 (92.9)	38 (90.5)	0.15	0.695
- Urinary Catheter	88 (95.7)	82 (97.6)	0.48	0.49	0.11	40 (95.2)	41 (97.6)	0.34	0.558
Microbiology & Exposure									
XDR Strain, n (%)	60 (65.2)	58 (69.0)	0.29	0.59	0.08	28 (66.7)	29 (69.0)	0.06	0.811
Multisite Carriage, n (%)			6.54	0.038	0.33			0.35	0.838
- Non-colonized	25 (27.2)	15 (17.9)				9 (21.4)	8 (19.0)		
- Single-site	37 (40.2)	28 (33.3)				15 (35.7)	16 (38.1)		
- Multisite (≥2 sites)	30 (32.6)	41 (48.8)				18 (42.9)	18 (42.9)		
Prior Antibiotic Use (90d), n (%)	75 (81.5)	72 (85.7)	0.56	0.455	0.11	35 (83.3)	36 (85.7)	0.09	0.763
- Meropenem/Imipenem	48 (52.2)	56 (66.7)	3.82	0.05	0.3	25 (59.5)	26 (61.9)	0.05	0.822
Physiology & Labs									
eGFR, mL/min/1.73m²	76.2 ± 28.5	58.4 ± 24.1	4.45	<0.001	0.67	64.5 ± 25.2	62.8 ± 26.1	0.3	0.762
Serum Albumin, g/L	30.2 ± 5.4	28.5 ± 6.1	1.96	0.052	0.29	29.1 ± 5.6	28.9 ± 5.8	0.16	0.874

Data are presented as mean ± standard deviation (SD) for continuous variables and n (%) for categorical variables. Group A: Colistin-Sulbactam; Group B: Colistin-Tigecycline. SMD: Standardized Mean Difference. For pre-matched comparisons, independent sample t-tests were used for continuous variables, and Pearson’s χ2 tests or Mann-Whitney U tests for categorical variables. For post-matched comparisons (paired data), paired t-tests and McNemar’s tests were utilized. An SMD < 0.10 indicates negligible imbalance between groups.

### 3.2 Analysis of predictors for microbiological eradication in the matched cohort

Multivariate logistic regression identified independent predictors of microbiological eradication in the propensity score-matched cohort (n = 84). Overall, 58 patients (69.0%) achieved eradication. Univariate analysis associated higher APACHE II scores, multisite colonization (≥2 sites), prior meropenem exposure, and the Colistin-Tigecycline regimen with a decreased likelihood of clearance.

Variables with P < 0.10 entered the multivariate model, confirming four independent predictors. Multisite colonization was a strong negative predictor (aOR 0.29; 95% CI 0.11–0.76; P = 0.012). Similarly, prior meropenem exposure (aOR 0.36; 95% CI 0.14–0.94; P = 0.037) and higher APACHE II scores (aOR 0.88; 95% CI 0.80–0.96; P = 0.005) independently hindered eradication. Furthermore, the treatment regimen remained an independent determinant: receiving Colistin-Tigecycline was associated with significantly lower odds of eradication compared to Colistin-Sulbactam (aOR 0.31; 95% CI 0.11–0.86; P = 0.025), reinforcing high-dose sulbactam’s greater microbiological clearance potential. **Table 2** presents detailed results.

**Table 2:** Univariate and multivariate logistic regression analysis for predictors associated with microbiological eradication in the matched cohort (n = 84).

Variable	Microbiological eradication (n=58)	Non-eradication (n=26)	Univariate analysis OR (95% CI)	P-value	Multivariate analysis aOR (95% CI)	P-value
Demographics						
Age, years	65.1 ± 12.0	67.5 ± 12.8	0.98 (0.95–1.02)	0.381	—	—
Male sex	37 (63.8%)	18 (69.2%)	0.78 (0.30–2.03)	0.622	—	—
Clinical Severity						
APACHE II Score	22.1 ± 5.2	26.1 ± 5.9	0.86 (0.79–0.95)	0.003	0.88 (0.80–0.96)	0.005
Septic Shock	28 (48.3%)	17 (65.4%)	0.49 (0.19–1.26)	0.141	—	—
Microbiology & Exposure						
XDR Strain	37 (63.8%)	20 (76.9%)	0.53 (0.19–1.48)	0.225	—	—
Multisite Colonization						
- Non-colonized	15 (25.9%)	2 (7.7%)	Reference	—	Reference	—
- Single-site	23 (39.7%)	8 (30.8%)	0.38 (0.07–2.02)	0.258	0.45 (0.08–2.53)	0.365
- Multisite (≥2 sites)	20 (34.5%)	16 (61.5%)	0.17 (0.03–0.86)	0.032	0.29 (0.11–0.76)	0.012
Prior Meropenem Exposure	31 (53.4%)	20 (76.9%)	0.35 (0.13–0.95)	0.039	0.36 (0.14–0.94)	0.037
Treatment Group						
Colistin-Tigecycline	24 (41.4%)	18 (69.2%)	0.31 (0.12–0.83)	0.019	0.31 (0.11–0.86)	0.025

Data are presented as mean ± SD or n (%). OR: Odds Ratio; aOR: adjusted Odds Ratio; CI: Confidence Interval. Variables with P < 0.10 in the univariate analysis were included in the multivariate model. The Hosmer-Lemeshow test indicated good model fit (P = 0.745). The reference group for treatment was Colistin-Sulbactam. In this model evaluating Eradication, an OR < 1 indicates a decreased likelihood of achieving microbiological clearance.

### 3.3 Primary outcome: microbiological eradication

**Figure 2:**
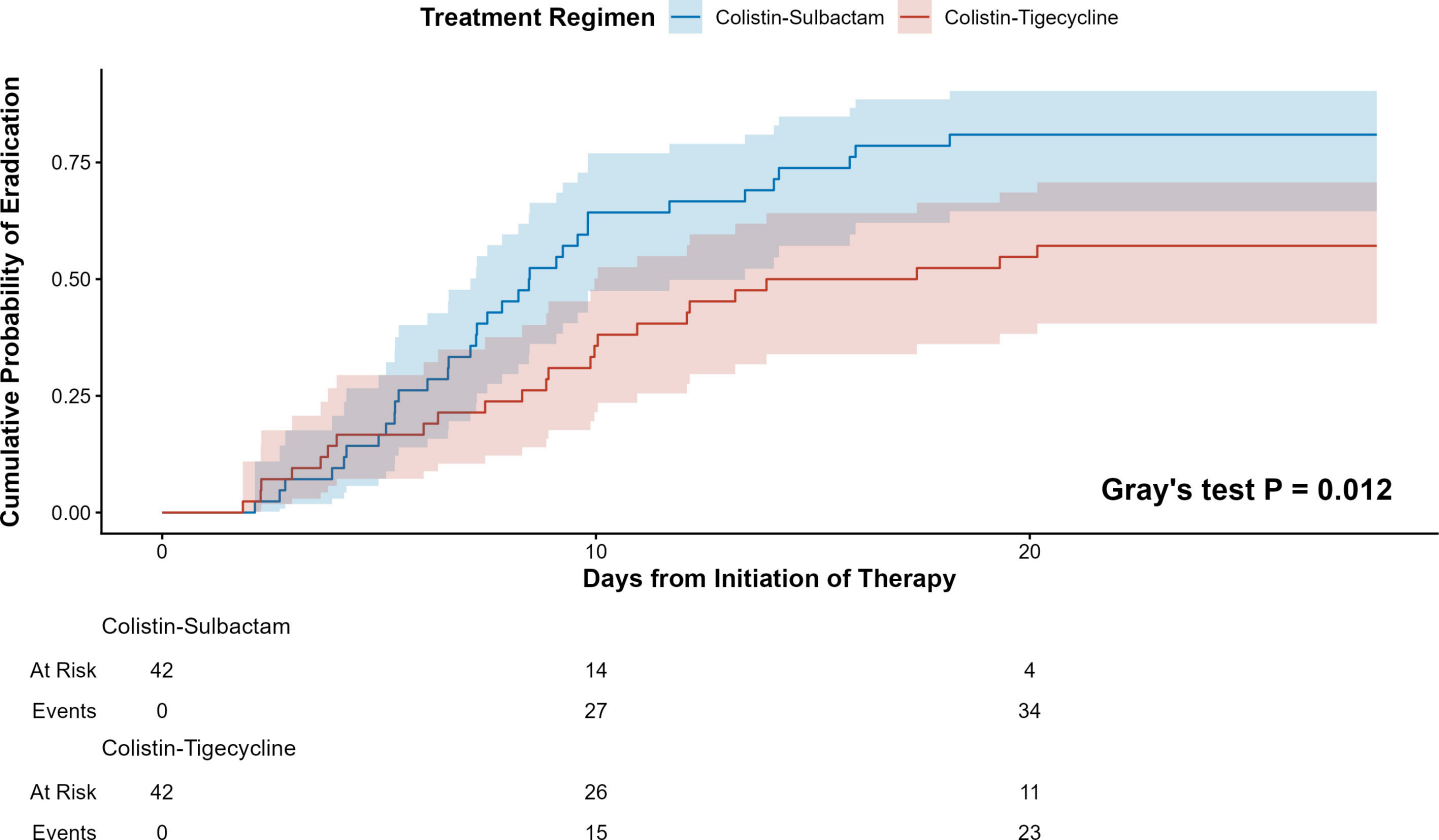
Cumulative incidence function (CIF) curves for time to microbiological eradication. The analysis was performed in the propensity score-matched cohort (n=84), strictly accounting for all-cause mortality as a competing risk. The cumulative probability of eradication was significantly higher in the Colistin-Sulbactam group compared to the Colistin-Tigecycline group (Gray’s test P = 0.012). Shaded areas represent 95% confidence intervals.

The primary endpoint of microbiological eradication was evaluated in the matched cohort (n = 84). Importantly, all 84 patients in this post-matched cohort had available follow-up respiratory tract cultures within the predefined time windows (or were statistically accounted for via the competing risk of mortality), thereby strictly constituting the microbiologically evaluable population. The analysis revealed a statistically significant advantage for the Colistin-Sulbactam regimen in achieving early microbiological clearance. At Day 7 (window: Days 5–9), the rate of microbiological eradication was significantly higher in Group A (Colistin-Sulbactam) compared to Group B (Colistin-Tigecycline) (66.7% vs. 40.5%; OR 2.94; 95% CI 1.25–6.92; P = 0.013). This advantage persisted through the test-of-cure visit at Day 14 (window: Days 12–16), where Group A maintained a higher eradication rate compared to Group B (81.0% vs. 57.1%; P = 0.018). Furthermore, recognizing that early mortality acts as a competing event that prevents the achievement of microbiological eradication, a competing risk analysis was performed. When accounting for all-cause mortality as a competing risk, the cumulative incidence of microbiological eradication remained significantly higher in the Colistin-Sulbactam group compared to the Colistin-Tigecycline group (Gray’s test P = 0.012) (**Figure 2**). Conversely, microbiological failure (persistence or recurrence of the original strain) was more frequently observed in Group B (42.9% vs. 19.0%; P = 0.018). The detailed microbiological outcomes are summarized in **Table 3**.

**Table 3:** Comparison of microbiological outcomes between matched groups (microbiologically evaluable population, n = 42 pairs).

Outcome measure	Group A (Col-Sul) (n=42)	Group B (Col-Tig) (n=42)	Difference (95% CI)	OR (95% CI)	P-value
Primary Endpoint					
Day 7 Eradication, n (%)	28 (66.7%)	17 (40.5%)	26.2% (5.8 to 46.6)	2.94 (1.25–6.92)	0.013
Day 14 Eradication, n (%)	34 (81.0%)	24 (57.1%)	23.9% (5.1 to 42.7)	3.17 (1.21–8.32)*	0.018
Microbiological Failure by Day 14, n (%)	8 (19.0%)	18 (42.9%)	-23.9% (-42.7 to -5.1)	0.32 (0.12–0.83)*	0.018
Persistence	6 (14.3%)	15 (35.7%)			
Recurrence	2 (4.8%)	3 (7.1%)			

CI, confidence interval; IQR, interquartile range; OR, odds ratio. All 84 matched patients had available follow-up cultures and thus qualified for the microbiologically evaluable population. The time-to-event analysis accounting for the competing risk of mortality is presented as a Cumulative Incidence Function in **Figure 2** (Gray’s test P = 0.012).

*Odds ratio calculated for Group A vs. Group B. All P-values in this table are from McNemar’s test for paired binary outcomes.

### 3.4 Secondary outcome: clinical efficacy and survival analysis

**Figure 3:**
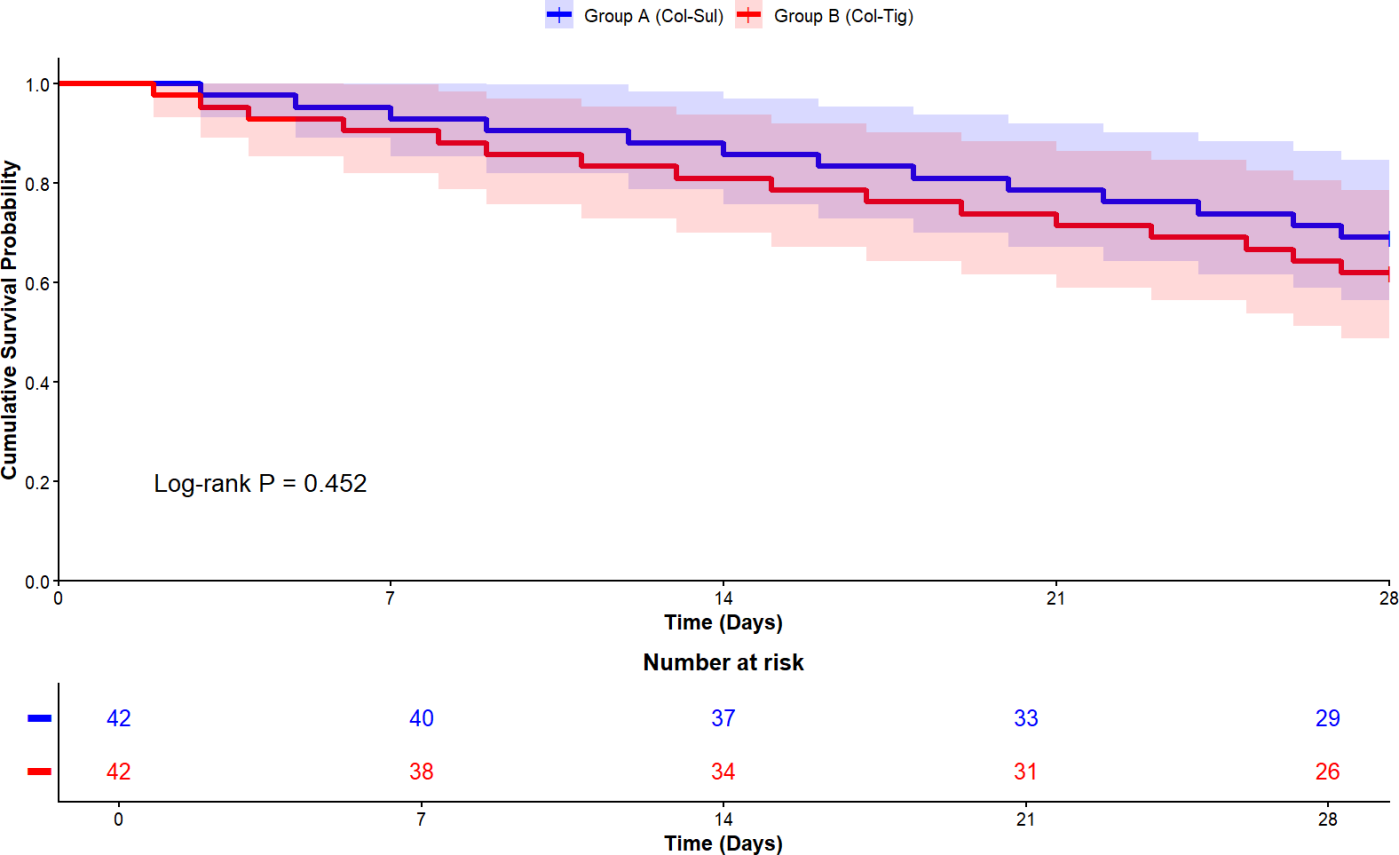
Kaplan-Meier estimates of 28-day all-cause mortality in the propensity score-matched cohort. The survival curves illustrate the cumulative probability of survival over 28 days for patients treated with Colistin-Sulbactam (Group A, blue line; n=42) versus Colistin-Tigecycline (Group B, red line; n=42). Shaded areas represent the 95% confidence intervals. The number of patients at risk at each 7-day interval is displayed below the X-axis.

Within the propensity score-matched cohort (n = 84), while the sulbactam-based regimen demonstrated higher microbiological eradication, this benefit translated only partially into clinical outcomes. In terms of clinical cure, Group A (Colistin-Sulbactam) exhibited a significantly higher cure rate compared to Group B (Colistin-Tigecycline) at the end of treatment (59.5% vs. 35.7%; P = 0.029), driven largely by the resolution of inflammatory markers and improvement in oxygenation. However, regarding the hard endpoint of survival, no statistically significant difference was observed between the two strategies. The 28-day all-cause mortality rate was 31.0% (13/42) in Group A and 38.1% (16/42) in Group B (Risk Difference -7.1%; 95% CI -26.8% to 12.5%; P = 0.490). The Kaplan-Meier survival analysis (**Figure 3**) further corroborated this finding. The survival curves for the two groups separated slightly during the first week but subsequently converged and overlapped, with the Log-rank test indicating no significant difference in overall survival probability over the 28-day observation period (Log-rank P = 0.452). Additionally, there were no significant differences in the overall hospital length of stay (LOS) or ICU stay between the two groups. Although the median ICU LOS was numerically lower in Group A (14.0 vs. 17.5 days), this difference did not reach statistical significance (P = 0.147) (**Table 4**).

**Table 4:** Secondary clinical outcomes, healthcare resource utilization, and safety profile in the matched cohort (n = 42 pairs).

Outcome measure	Group A (Col-Sul) (n = 42)	Group B (Col-Tig) (n = 42)	Difference (95% CI)	P-value
Survival Endpoint				
28-Day All-Cause Mortality, n (%)	13 (31.0%)	16 (38.1%)	RD: -7.1% (-26.8% to 12.5%)	0.49
Clinical Efficacy				
Clinical Cure, n (%)	25 (59.5%)	15 (35.7%)	RD: 23.8% (2.9% to 44.7%)	0.029
Treatment Failure (Composite), n (%)	15 (35.7%)	24 (57.1%)	RD: -21.4% (-42.1% to -0.7%)	0.048
Resource Utilization				
Hospital LOS, days, median (IQR)	28.0 (18.0–45.0)	31.0 (20.0–49.0)	—	0.399
ICU LOS, days, median (IQR)	14.0 (9.0–24.0)	17.5 (10.0–29.0)	—	0.147
Nephrotoxicity (KDIGO)				
Overall AKI, n (%)	14 (33.3%)	16 (38.1%)	RD: -4.8% (-25.3% to 15.7%)	0.648
AKI Severity Stage, n (%)			—	0.950†
- Stage 1	8 (19.0%)	8 (19.0%)		
- Stage 2	4 (9.5%)	5 (11.9%)		
- Stage 3	2 (4.8%)	3 (7.1%)		
Requirement for RRT, n (%)	1 (2.4%)	2 (4.8%)	RD: -2.4% (-13.5% to 8.7%)	1
Other Adverse Events				
Hepatotoxicity, n (%)	2 (4.8%)	7 (16.7%)	RD: -11.9% (-26.5% to 2.7%)	0.18
Neurotoxicity, n (%)	1 (2.4%)	1 (2.4%)	RD: 0.0% (-9.3% to 9.3%)	1
Discontinuation due to AE, n (%)	1 (2.4%)	3 (7.1%)	RD: -4.8% (-16.2% to 6.6%)	0.625

LOS: Length of Stay; ICU: Intensive Care Unit; RD: Risk Difference (Group A minus Group B); CI: Confidence Interval; AKI: Acute Kidney Injury; RRT: Renal Replacement Therapy; AE: Adverse Event; ULN: Upper Limit of Normal. P-values for binary outcomes were calculated using McNemar’s test for paired comparisons. P-values for continuous variables (LOS) were calculated using the Wilcoxon signed-rank test. †P-value for the distribution of AKI stages was derived from the Marginal Homogeneity Test. Clinical Cure was assessed at the end of treatment or discharge. Treatment Failure is the composite endpoint defined in the Methods section. Hepatotoxicity was defined as ALT or AST >3× ULN.

### 3.5 Safety assessment: nephrotoxicity and other adverse events

In the matched cohort, overall AKI incidence was high but showed no significant difference between groups (**Table 4**). AKI rates were 33.3% (14/42) in the Colistin-Sulbactam group versus 38.1% (16/42) in the Colistin-Tigecycline group (RD -4.8%; 95% CI -25.3% to 15.7%; P = 0.648). Severity was comparable (P = 0.950), primarily involving Stage 1 or 2. Stage 3 AKI (4.8% vs. 7.1%) and new-onset RRT (2.4% vs. 4.8%; P = 1.000) were infrequent. Regarding non-renal events, hepatotoxicity occurred in 4.8% of Group A versus 16.7% of Group B (RD -11.9%; 95% CI -26.5% to 2.7%; P = 0.180). Neurotoxicity was rare and evenly distributed. Discontinuation due to adverse events occurred in one patient in Group A and three in Group B.

### 3.6 Subgroup analysis for XDR strains and bacterial burden

**Figure 4:**
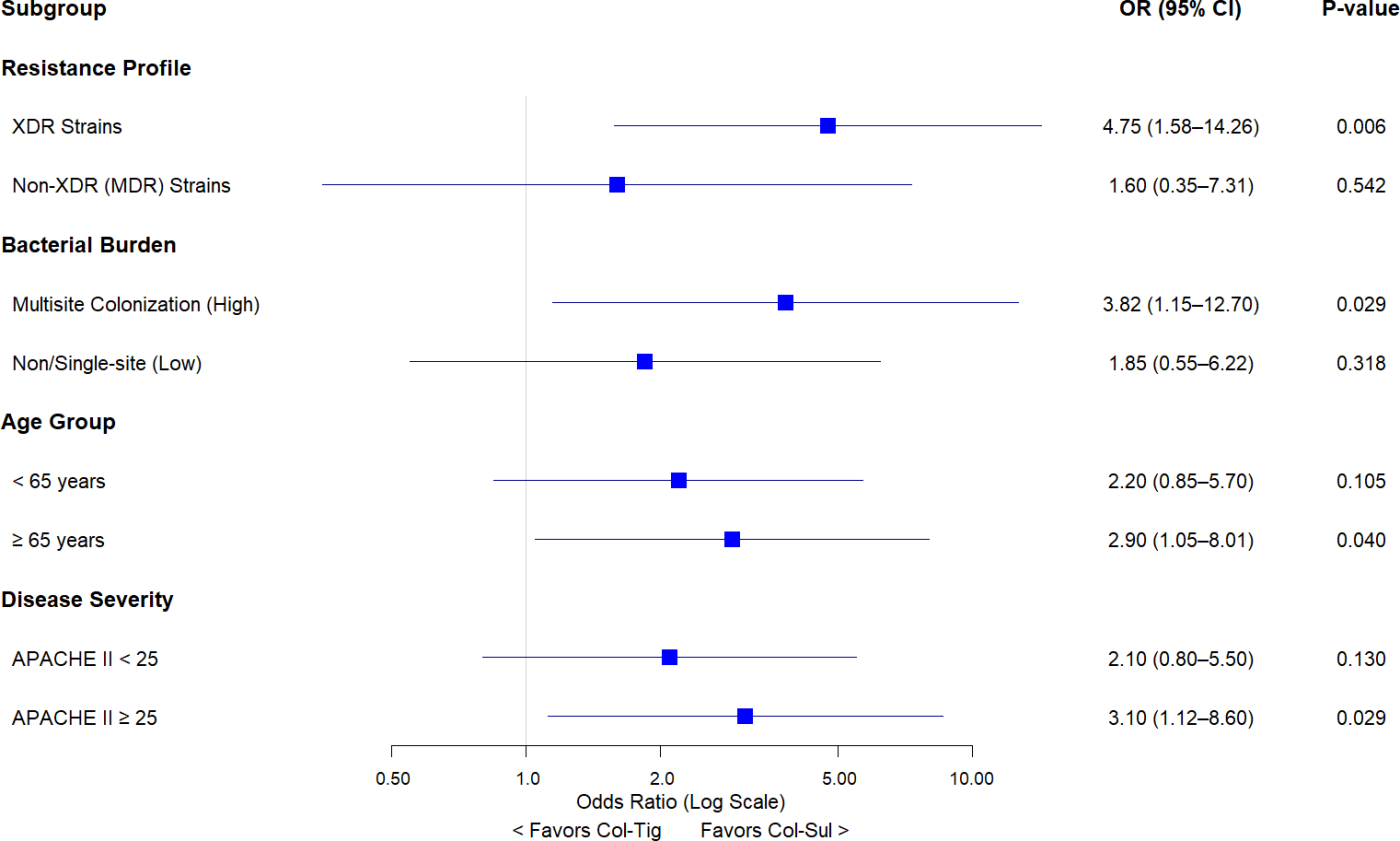
Forest plot of subgroup analysis for day 7 microbiological eradication. The plot displays the Odds Ratios (OR) and 95% Confidence Intervals (CI) for microbiological eradication, favoring the Colistin-Sulbactam group (right side) over the Colistin-Tigecycline group (left side). The vertical line at OR = 1 represents no treatment effect. The analysis demonstrates that the efficacy gap between the two regimens is widest in “hard-to-treat” populations, specifically those with XDR strains (OR 4.75; 95% CI 1.58–14.26) and high baseline bacterial burden (Multisite Colonization) (OR 3.82; 95% CI 1.15–12.70). P-values indicate the significance of the treatment effect within each subgroup calculated using logistic regression interaction terms.

Subgroup analysis evaluated treatment consistency across clinical profiles (**Figure 4**). In the XDR A. baumannii cohort, representing most matched patients (Group A: 66.7%; Group B: 69.0%), Colistin-Sulbactam demonstrated pronounced advantage. For XDR strains, Day 7 eradication reached 71.4% (20/28) in Group A versus 34.5% (10/29) in Group B (OR 4.75; 95% CI 1.58–14.26; P = 0.006), while the difference for non-XDR strains was less marked (OR 1.60; 95% CI 0.35–7.31; P = 0.542). Stratification by bacterial burden revealed an amplified benefit for the sulbactam-based regimen in multisite colonization (≥2 sites) (OR 3.82; 95% CI 1.15–12.70; P = 0.029). Conversely, for patients with lower burden (non-colonized or single-site), the effect size attenuated and was not statistically significant (OR 1.85; 95% CI 0.55–6.22; P = 0.318). Other subgroups stratified by age and APACHE II score demonstrated consistent trends favoring Group A, with no significant treatment-by-subgroup interactions detected (all P for interaction > 0.10).

## 4 Discussion

In this study, we found that a polymyxin-sulbactam combination regimen resulted in higher microbiological eradication and improved clinical cure rates compared to a polymyxin-tigecycline regimen for the treatment of CRAB HAP/VAP. Notably, this microbiological advantage was most pronounced in patients with XDR strains and high bacterial burden (multisite colonization). Our findings contribute to the growing body of evidence supporting the use of high-dose sulbactam as a cornerstone in the management of these severe infections. The observed advantage can be understood through a multi-faceted analysis of the drugs’ pharmacokinetic and pharmacodynamic (PK/PD) properties, their impact on the host-pathogen interaction, and their respective safety profiles. The distinction between the bactericidal nature of sulbactam and the bacteriostatic action of tigecycline is of paramount importance in the context of life-threatening infections such as HAP/VAP (Huang et al., 2024). While high-dose tigecycline achieves excellent penetration into the pulmonary epithelial lining fluid (ELF), its bacteriostatic mechanism may be insufficient to achieve rapid and complete bacterial eradication in critically ill or immunocompromised patients (Dimopoulos et al., 2022). Conversely, sulbactam exerts intrinsic bactericidal activity against *A. baumannii*, and modern dosing strategies using high doses (e.g., 9g daily) with extended infusions are designed to maximize the pharmacodynamic index, the percentage of the dosing interval that free drug concentrations remain above the minimum inhibitory concentration (%fT>MIC). This approach has been shown to achieve the necessary PK/PD targets to be effective against strains with MICs up to 8 mg/L, which are often classified as intermediately susceptible (Abouelhassan et al., 2024). The latest Infectious Diseases Society of America (IDSA) guidance reflects this, prioritizing sulbactam-based regimens and relegating tigecycline to alternative status for non-bacteremic infections (Infectious Diseases Society of America, 2024). Our results align with this guidance, suggesting the bactericidal effect of sulbactam translates to improved clinical efficacy in severe pneumonia.

Beyond the local infection in the lungs, the concept of a ‘total bacterial burden’ is critical for understanding treatment outcomes in CRAB infections. *A. baumannii* is known to colonize multiple body sites, including the gut and skin, creating endogenous reservoirs that can fuel recurrent infections and increase the risk of mortality (Peghin et al., 2024). A bactericidal agent like sulbactam may be more effective than a bacteriostatic one at suppressing these reservoirs, thereby reducing the total bacterial load and preventing the reseeding of infection into the lungs or bloodstream (Boutzoukas and Doi, 2025). A key finding of our study is the independent prognostic value of multisite colonization. Our multivariate analysis identified colonization at ≥2 non-respiratory sites as a strong independent predictor of treatment failure. This observation is consistent with recent prospective evidence demonstrating that multisite colonization is a robust independent predictor for the development of subsequent CRAB infections (Cogliati Dezza et al., 2023) and bloodstream infections (Cogliati Dezza et al., 2026) in colonized critically ill patients. Furthermore, our subgroup analysis revealed that the benefit of the sulbactam regimen was amplified in this high-burden population. This suggests that a bactericidal agent like high-dose sulbactam may be more effective than a bacteriostatic one at suppressing this total body bacterial load, thereby preventing the reseeding of infection. This highlights that successful treatment of CRAB-HAP may require an approach that addresses the pathogen not just as a pulmonary invader, but as a systemic colonizer.

Our study must also be interpreted in the context of the well-documented dissociation between microbiological clearance and clinical survival in severe CRAB infections. The phenomenon of ‘competing risks’ is central to this issue, where the patient’s underlying severity of illness and state of sepsis-induced immunosuppression often become more significant drivers of mortality than the infection itself (Frattari et al., 2024). In patients with high APACHE II scores and multi-organ dysfunction, successful bacterial eradication may not be sufficient to alter the trajectory toward death if irreversible organ damage has already occurred or if the host is in a state of ‘immunoparalysis’, characterized by markers like persistent lymphocytopenia (Frattari et al., 2024). Furthermore, CRAB possesses a formidable arsenal of virulence factors, including the ability to form biofilms and persister cells, which allow it to evade both host immunity and antibiotic action (Shadan et al., 2023). These mechanisms can lead to a state where standard cultures appear negative, yet a persistent, low-level infection continues to provoke systemic inflammation or local tissue damage, contributing to poor outcomes. This complex interplay underscores that while an effective antibiotic regimen is necessary, it is only one component of managing these critically ill patients.

One of the most significant findings of our research is the favorable safety profile of the polymyxin-sulbactam combination, particularly concerning nephrotoxicity. The use of polymyxins is notoriously limited by their potential for acute kidney injury. Our results are consistent with and reinforce previous evidence suggesting that the co-administration of high-dose sulbactam does not exacerbate, and may in fact mitigate, polymyxin-associated nephrotoxicity (Saelim et al., 2021). This is a finding of major clinical importance. The recent pivotal trial for sulbactam-durlobactam provided strong support for this concept, demonstrating a substantially lower rate of nephrotoxicity compared to colistin-based therapy (13% vs. 38%) (Zhang et al., 2024). In contrast to the favorable renal profile, we observed a higher incidence of hepatotoxicity in the tigecycline group (16.7% vs. 4.8%), consistent with the known side effect profile of tetracycline derivatives. This renal-sparing potential of sulbactam, combined with a lower risk of liver injury, positions it as a safer partner for polymyxins in combination therapy. To further optimize both safety and efficacy, the implementation of therapeutic drug monitoring (TDM) is essential. International consensus guidelines strongly advocate for TDM for polymyxins to maintain an AUCss, 24hr of approximately 50 mg·h/L, thereby balancing antibacterial effect against the risk of toxicity (Tsuji et al., 2019). Similarly, TDM for high-dose sulbactam is warranted, particularly in patients with renal dysfunction or augmented renal clearance, to ensure that PK/PD targets are met without unnecessary drug accumulation (Charoenwong et al., 2025). The lack of universal TDM represents a significant pharmacological limitation, as both colistin and sulbactam exhibit profound PK/PD variability in the critically ill. Factors such as augmented renal clearance (ARC) or fluctuating volumes of distribution in septic shock can lead to unpredictable drug exposure. Without real-time TDM data, it remains uncertain whether optimal therapeutic targets were consistently achieved in either cohort, which could potentially obscure dose-dependent efficacy or exacerbate unrecognized toxicity. The innovation of our study lies not only in the head-to-head comparison but also in highlighting a pragmatic regimen that can potentially maximize efficacy while actively enhancing patient safety.

This study has several limitations. As a retrospective analysis, it is susceptible to selection bias and unmeasured confounders that may have influenced treatment allocation and outcomes. The sample size may limit the statistical power to detect smaller differences between the groups. We utilized available clinical cultures to assess colonization rather than a systematic active surveillance protocol across all hospital departments. This discrepancy in institutional policy may introduce selection bias, as critically ill ICU patients undergo routine screening, whereas cultures for non-critically ill patients in general wards are predominantly symptom-driven, potentially underestimating the true burden of multisite colonization in the latter group. Additionally, the lack of biomarkers for immune status means our discussions on immunoparalysis remain mechanistic hypotheses requiring further investigation. Moreover, therapeutic drug monitoring was not universally implemented, meaning some patients in either group may have had suboptimal drug exposure. Crucially, patients in the tigecycline arm received a standard dosing regimen (100 mg loading, followed by 50 mg every 12 hours) rather than the high-dose strategy (e.g., 200 mg loading, 100 mg every 12 hours) that is increasingly recommended for severe CRAB infections, particularly HAP/VAP, to overcome its poor pulmonary penetration. This standard dosing may have led to suboptimal lung tissue concentrations, thereby contributing to the observed inferior efficacy in the tigecycline group. The persistent utilization of this standard dosage at our institution during the study period, despite the FDA warning regarding increased mortality in VAP, was primarily driven by strict adherence to approved product labeling and significant clinical apprehension regarding dose-dependent toxicities, particularly severe coagulopathy (e.g., hypofibrinogenemia) associated with off-label high-dose tigecycline. The heterogeneity in clinical practice, including variations in exact CMS dosing adaptations based on fluctuating renal function, represents a potential confounding factor. Furthermore, our strict inclusion of only CMS-treated patients to ensure pharmacokinetic consistency means our findings may not be fully generalizable to institutions predominantly utilizing other polymyxins, such as polymyxin B or colistin sulfate.

Furthermore, it is imperative to acknowledge that the treatment landscape for CRAB is rapidly evolving. Newer antibiotics, specifically novel siderophore cephalosporins like cefiderocol (Gatti et al., 2024) and targeted beta-lactamase inhibitor combinations like sulbactam-durlobactam (Kaye et al., 2023), have recently demonstrated potent *in vitro* and *in vivo* efficacy against CRAB and may eventually supersede older salvage therapies. However, as these novel agents were largely unavailable or restricted at our institution during the study timeframe, optimizing existing polymyxin-based combinations remains highly relevant for current global practice, particularly in resource-constrained settings.

## 5 Conclusion

This study suggests that a combination of a polymyxin with high-dose sulbactam is associated with significantly higher microbiological clearance and clinical cure rates compared to a polymyxin-tigecycline regimen for patients with CRAB-HAP/VAP, particularly for those with XDR strains or multisite colonization. However, it is critical to note that these microbiological advantages did not translate into a significant 28-day survival benefit in our cohort. This finding is supported by the favorable pharmacodynamic profile and renal-protective properties of sulbactam. Looking forward, there is a clear need for large-scale, prospective randomized controlled trials to confirm these findings. Future studies should incorporate systematic screening for multisite colonization, routine therapeutic drug monitoring for all agents, and the analysis of host immune biomarkers to better stratify patients and personalize therapy. By integrating these elements, we can move closer to optimizing treatment for this challenging pathogen and improving the prognosis for critically ill patients.

## Data Availability

The original contributions presented in the study are included in the article/supplementary material. Further inquiries can be directed to the corresponding author.
